# Molecular engineering and plant expression of an immunoglobulin heavy chain scaffold for delivery of a dengue vaccine candidate

**DOI:** 10.1111/pbi.12741

**Published:** 2017-07-15

**Authors:** Mi‐Young Kim, Craig Van Dolleweerd, Alastair Copland, Matthew John Paul, Sven Hofmann, Gina R. Webster, Emily Julik, Ivonne Ceballos‐Olvera, Jorge Reyes‐del Valle, Moon‐Sik Yang, Yong‐Suk Jang, Rajko Reljic, Julian K. Ma

**Affiliations:** ^1^ Institute for Infection and Immunity St George's University of London London UK; ^2^ Department of Molecular Biology and The Institute for Molecular Biology and Genetics Chonbuk National University Jeonju Korea; ^3^ School of Life Sciences Arizona State University Tempe AZ USA

**Keywords:** plant biotechnology, immunoglobulin G, dengue, vaccine, infection

## Abstract

In order to enhance vaccine uptake by the immune cells *in vivo*, molecular engineering approach was employed to construct a polymeric immunoglobulin G scaffold (PIGS) that incorporates multiple copies of an antigen and targets the Fc gamma receptors on antigen‐presenting cells. These self‐adjuvanting immunogens were tested in the context of dengue infection, for which there is currently no globally licensed vaccine yet. Thus, the consensus domain III sequence (cEDIII) of dengue glycoprotein E was incorporated into PIGS and expressed in both tobacco plants and Chinese Ovary Hamster cells. Purified mouse and human cEDIII‐PIGS were fractionated by HPLC into low and high molecular weight forms, corresponding to monomers, dimers and polymers. cEDIII‐PIGS were shown to retain important Fc receptor functions associated with immunoglobulins, including binding to C1q component of the complement and the low affinity Fcγ receptor II, as well as to macrophage cells *in vitro*. These molecules were shown to be immunogenic in mice, with or without an adjuvant, inducing a high level IgG antibody response which showed a neutralizing potential against the dengue virus serotype 2. The cEDIII‐PIGS also induced a significant cellular immune response, IFN‐γ production and polyfunctional T cells in both the CD4^+^ and CD8^+^ compartments. This proof‐of‐principle study shows that the potent antibody Fc‐mediated cellular functions can be harnessed to improve vaccine design, underscoring the potential of this technology to induce and modulate a broad‐ranging immune response.

## Introduction

As the development of safe and potent adjuvants has been a major bottleneck in vaccine development against many diseases, our focus in recent years has been to generate adjuvanticity by molecular engineering, and in that way minimize the vaccine's reliance on powerful exogenous adjuvants that may have undesirable toxic effects. Such self‐adjuvanting vaccines might be sufficiently immunogenic on their own or could be applied with currently available, licensed adjuvants such as alum. Here, we employed an approach based on a polymeric IgG‐Fc fusion protein (Mekhaiel *et al*., 2011; Smith and Morrison, 1994) to design an immune complex‐like molecule modelled on pentameric/hexameric IgM, for efficient delivery of multiple copies of consensus domain III (Leng *et al*., 2009) of the dengue envelope glycoprotein to Fc‐gamma bearing antigen‐presenting cells (APC). Dengue epidemic requires urgently a cheap and globally applicable vaccine, as there are around 400 million people around the world currently infected, while almost half of the human population is at risk (Lam, 2013). Despite substantial efforts to develop a dengue vaccine in recent years (Beckett *et al*., 2011; Coller and Clements, 2011; Danko *et al*., 2011; Guy *et al*., 2011; Lindow *et al*., 2013; Murphy and Whitehead, 2011; Osorio *et al*., 2011), progress has been frustratingly slow. An important complication has been the need to induce protective immunity against all four known serotypes of the dengue virus, while circumventing the issue of serotype cross‐immunity, which can lead to antibody‐dependent enhancement (ADE) of infection (Balsitis *et al*., 2010; Chau *et al*., 2008; Kliks *et al*., 1988; Moi *et al*., 2013). The latter phenomenon occurs during both natural infection and suboptimal vaccine‐induced immune responses and can lead to more severe manifestations of the subsequent infection. The Dengvaxia vaccine developed by Sanofi Pasteur (Schwartz *et al*., 2015) and based on attenuated yellow fever virus performed sufficiently well in two large efficacy trials in South‐East Asia and Latin America (Capeding *et al*., 2014; Villar *et al*., 2015), and in the three‐year follow‐up studies (Hadinegoro *et al*., 2015) to warrant its recent licensure in Mexico, Brazil and Philippines. However, the vaccine was less effective in children younger than 9 years. Furthermore, the follow‐up studies suggested that the incidence and severity of dengue infection were worse in children from the vaccinated than the control group, most likely due to ADE. Coupled with the relatively poor protection against the DENV2 serotype, this clearly illustrates the difficulties and challenges for developing a universal dengue vaccine. In fact, it may well be that an effective dengue vaccination strategy may require multiple vaccines aimed at different age groups or populations.

To minimize the risk of ADE, the target of many new vaccine approaches has been the domain III of the viral envelope glycoprotein E, which induces relatively lower levels of non‐neutralizing antibodies than the other domains (Guzman *et al*., 2010). Mouse sera induced by a DIII‐based DNA vaccine were also highly reactive to infective viral particles in a virus‐capture ELISA and specific for each serotype as revealed by the low cross‐reactive and cross‐neutralizing activities (Poggianella *et al*., 2015). The serotype‐specific sera did not induce antibody dependent enhancement of infection (ADE) in non‐homologous virus serotypes. Furthermore, it has been previously shown that the consensus domain III (cEDIII) can induce antibodies to all four serotypes and therefore simplify vaccine design considerably (Leng *et al*., 2009), dispensing with the need for a tetravalent vaccine, with its attendant issues of complexity of production and serotype interference. cEDIII was originally generated by aligning the EDIII sequences from each of the four serotypes in five clinical isolates for each serotype (Leng *et al*., 2009). Unfortunately, domain III is notoriously poorly immunogenic and requires powerful adjuvants to induce neutralizing antibodies (Chen *et al*., 2013; Chiang *et al*., 2011, 2014; Harahap‐Carrillo *et al*., 2015; Hermida *et al*., 2006). Here, we set out to test whether the PIGS bioengineering approach could improve the immunogenicity of cEDIII and induce a broad immune response to this antigen, which could then be harnessed in vaccine design for dengue infection. We chose to use both the plant and mammalian expression system, for comparison purposes, with the former also intended for potential scale‐up production of such molecules at comparatively lower costs.

## Results

### Genetic construction for cEDIII‐PIGS

**Figure 1:**
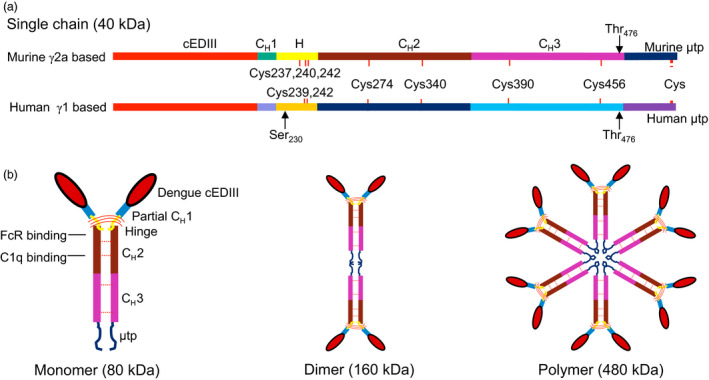
Construction of cEDIII‐PIGS. (a) Linearized map of murine γ2α and human γ1−based cEDIII‐PIGS sequence showing the key genetic elements; (b) schematic illustration of the basic cEDIII‐PIGS constituent (the ‘monomer’), and the higher molecular structures represented by the dimer and polymers (hexamers). The position of the dengue antigen in place of antibody variable region and the binding domains for C1q and Fc receptor are indicated in the monomer construct. The expected molecular weights of each construct are indicated.

The genes encoding the cEDIII‐PIGS single chain protein were cloned and expressed both in genetically modified *N. benthamiana* and mammalian CHO cells. Both mouse (based on IgG2a) and human (based on IgG1) version of these molecules were generated. The linear depiction of the cloned cEDIII‐PIGS sequence is shown in Figure 1a. cEDIII antigen replaced the variable region of the immunoglobulin γ chain molecule. The antigen is linked to Cγ2 and Cγ3 domains by a short peptide derived partly from Cγ1 domain and partly from the hinge region. The μtp is genetically linked at the C' terminus. Two amino acid substitutes were added: a serine at position 230 (human) to avoid an unpaired cysteine residue and a threonine at position 476 (murine and human) to mimic the C terminus of IgM CH4 domain. cEDIII‐PIGS are expected to form IgG‐like structures as shown in Figure 1b, with the dengue cEDIII antigen on the N terminal end and the IgM‐derived μ tail piece on the C terminal end. The latter can facilitate polymerization of the monomers to form dimers and higher polymers (i.e. hexamers), much like the IgM itself. Furthermore, if co‐expressed with the J chain, these molecules can form IgM‐like pentamers.

### Expression of cEDIII‐PIGS in plants and CHO cells

**Figure 2:**
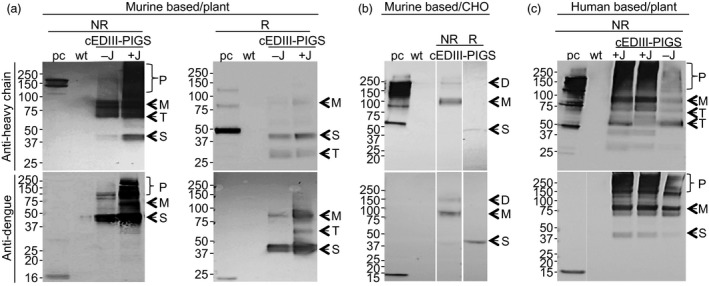
Expression of murine and human cEDIII‐PIGS. Western blot detection of murine (a), in plants; (b), in CHO cells) and human (c), in plants) cEDIII‐PIGS by anti‐Ig γ antiserum (upper panels) or by anti‐dengue (lower panels) monoclonal antibodies. Lanes: ‘pc’, (positive control) mouse IgG2a (a, b) or human IgG1 (c) (upper panels) or bacterially expressed recombinant cEDIII (lower panels); ‘wt’: wild‐type plant extract; −J: cEDIII‐PIGS expressed without J chain; +J: cEDIII‐PIGS expressed with J chain. The proteins were electrophoretically resolved under the non‐reducing (NR) or reducing (R) conditions. Indicated are the different molecular forms present in the cEDIII‐PIGS preparations: P—polymer; D—dimer; M—monomer; S—single chain; T—truncated forms. Molecular weight markers (in kDa) are shown on the left.

Mouse cEDIII‐PIGS molecules were expressed in *N. benthamiana* plants by infiltration of plant leaves with corresponding pTRAk.2 vector. After 6 days, the plant leaves were harvested, protein extract was prepared and analysed by SDS‐PAGE and Western blotting with detection by anti‐IgG heavy chain antiserum (Figure 2a upper panel) or anti‐dengue monoclonal antibody (Figure 2a, lower panel) under non‐reducing (NR) or reducing (R) conditions. Under non‐reducing conditions, the murine cEDIII‐PIGS presented as immunoreactive protein bands of varying sizes ranging from 40 to 80 kDa and several molecular forms of 160 kDa and above (Figure 2a, NR). In the presence of J chain (lane +J), there was a higher proportion of polymeric forms, when compared to constructs without J chain (lane −J). Consequently, the J chain version of the cEDIII‐PIGS derived from plant was used in the subsequent studies. Under reducing conditions, a predominant 40 kDa single chain polypeptide was observed (labelled ‘S’), with evidence of some degradation (i.e. the 28 kDa protein band). Wild‐type plant extracts (lane ‘wt’) and commercial mouse IgG2a (lane ‘pc’, upper panel) were used as the internal negative and positive controls, respectively. When probed with an anti‐dengue antibody (Figure 2a, lower panel), the specific protein profiles were very similar to those seen with anti‐IgG antibody (Figure 2a, upper panel), thus confirming the presence of the antigen within the cEDIII‐PIGS molecules. Wild‐type plant extract (lane ‘wt’) showed no immunoreactivity with the anti‐dengue antibody, while the bacterially expressed dengue cEDIII antigen (lane ‘pc’, lower panel) served as the positive control. The cEDIII‐PIGS were also expressed in CHO cells, and several cell clones were generated. A dominant protein band corresponding to monomer (80 kDa) was detected by both IgG antiserum and dengue antibody in the supernatant of transfected cells, while only a weak protein band was detected for the dimer (Figure 2b). We also expressed the human version of cEDIII‐PIGS in plants. Similar to mouse cEDIII‐PIGS, the presence of J chain increased the proportion of the polymeric forms detected with anti‐IgG heavy chain antiserum (Figure 2c, upper panel) or anti‐dengue antibody (lower panel). However, human cEDIII‐PIGS showed a significantly higher proportion of polymeric forms than the mouse cEDIII‐PIGS (lanes +J, two infiltrated plants). Human IgG1 served as the positive control while wild‐type plant extract was used as the negative control.

### Purification and HPLC analysis of cEDIII‐PIGS

**Figure 3:**
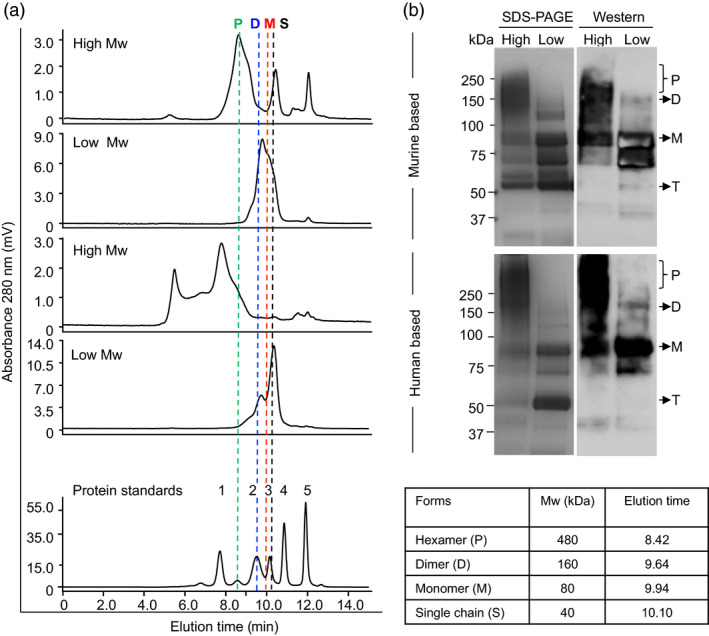
HPLC fractionation and molecular size analysis of purified mouse and human cEDIII‐PIGS. (a) HPLC profiles of separated low and high molecular weight mouse and human plant‐derived cEDIII‐PIGS fractions (50 μg); the accompanying table and the vertical doted lines indicate the expected molecular weights and retention times, respectively, of various molecular forms (i.e. P, polymer, D, dimer, M, monomer, S, single chain). The gel filtration standards (bottom panel) were 1. 670; 2.158; 3. 44; 4. 17 and 5. 1.35 kDa. (b) SDS‐PAGE and Western blot analysis of HPLC fractionated murine and human cEDIII‐PIGS under non‐reducing conditions. In addition to above‐mentioned molecular forms, both mouse and human cEDIII‐PIGS display a prominent 50 kDa protein band which is not immunoreactive with anti‐dengue antibody and is most likely a truncated (T) form.

Murine cEDIII‐PIGS molecules were purified from plant extracts or CHO cell culture supernatants using affinity chromatography. The final yields were 17 mg/kg fresh weight plant tissue, or 2.5 mg/L CHO culture supernatant, respectively. After concentration, typically to 1 mg/mL, plant‐derived murine and human cEDIII‐PIGS were subjected to HPLC fractionation of low and high molecular weight forms (Figure 3). Fractionated components were analysed by comparison with gel‐filtration protein standards (Figure 3a) and by SDS‐PAGE/Coomassie staining and Western blotting (Figure 3b). HPLC profiles indicated that high molecular weight fraction of mouse cEDIII‐PIGS contained a mixture of molecular species ranging from dimers (expected size 160 kDa) to polymers (expected size 480 kDa), although SDS‐PAGE and Western analysis (Figure 3b) revealed predominantly a diffuse 150–250 protein band likely corresponding to dimers and trimers, in addition to some truncated forms. The low molecular weight fraction contained a mixture of monomer (80 kDa) and various truncated forms (50–70 kDa), which could not be separated. Human cEDIII‐PIGS displayed exclusively polymers in the high molecular weight fraction, ranging from the expected 480–680 kDa, possibly representing pentamers/hexamers and some higher molecular conjugates, respectively. This fraction also contained large molecular aggregates (>1000 kDa). Low molecular weight fraction contained monomers and a truncated forms. SDS‐PAGE and Western blot analysis (Figure 3b) of the high molecular weight fraction of human cEDIII‐PIGS indicated the presence of a diffuse protein band of 250 kDa and above but the exact size could not be determined.

### Functional characterization of cEDIII‐PIGS

**Figure 4:**
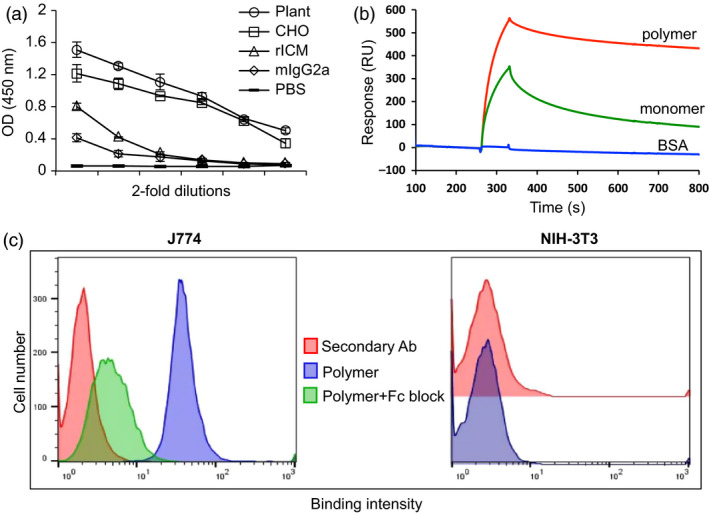
Functional characterization cEDIII‐PIGS and Fcγ receptor interaction. (a) cEDIII‐PIGS binding to C1q by ELISA; shown are ELISA readings for serial dilutions of cEDIII‐PIGS starting at 5 μg/mL); rICM = recombinant immune complex mimics (Pepponi *et al*., 2013) (used as a positive control). (b) Surface plasmon resonance (Biacore) comparative analysis of murine plant‐derived cEDIII‐PIGS fractions following HPLC separation (dimer/polymers *vs* monomer) binding to mouse low affinity FcγRII receptor (CD16); shown are the binding curves obtained with 10 μg/ml of cEDIII‐PIGS or an irrelevant protein (BSA) used as the negative control. (c) Cell binding and flow cytometry analysis of unfractionated plant derived cEDIII‐PIGS binding to macrophage J774 and fibroblast NIH‐3T3 cells, respectively. Red histograms indicate binding of the secondary antibody FITC conjugate alone, blue histograms show cEDIII‐PIGS binding and green histograms indicate cEDIII binding in the presence of anti‐CD16/32 antibody (‘Fc block’). Experiment performed two times with similar results.

To characterize these molecules functionally, the cEDIII‐PIGS were tested in three binding assays. In the first, their potential to bind the C1q component of complement cascade was tested by ELISA (Figure 4a). Both the plant and CHO‐derived unfractionated cEDIII‐PIGS bound to C1q in a concentration‐dependent manner. PBS and murine IgG2a used as the negative control did not bind, while ICM (immune complex mimics), which contains multiple copies of a murine IgG1 mAb bound to a polymeric *Mycobacterium tuberculosis* antigen (Pepponi *et al*., 2013), was used as the positive control. In the second assay, we tested the capacity of fractionated mouse polymeric and monomeric cEDIII‐PIGS to bind to the immobilized mouse FcγRII (CD16), in a Biacore assay. Polymeric cEDIII‐PIGS bound with a fast ‘on’ and slow ‘off’ rate, whereas the monomeric form bound less effectively to the same receptor (Figure 4b). Finally, in the third assay, we tested the capacity of cEDIII‐PIGS polymers to bind to Fcγ receptor positive (J774 macrophages) and negative (NIH‐3T3 fibroblasts) cells. Figure 4c shows the binding of cEDIII‐PIGS to J774 (blue histogram) but not to NIH‐3T3 cells, when compared to secondary antibody alone (red histogram). This binding could be reversed to a large degree by cell preincubation with the blocking anti‐CD16/32 antibodies, thus confirming the specificity of FcγR binding.

### ELISA analysis of IgG response to cEDIII‐PIGS in mice

**Figure 5:**
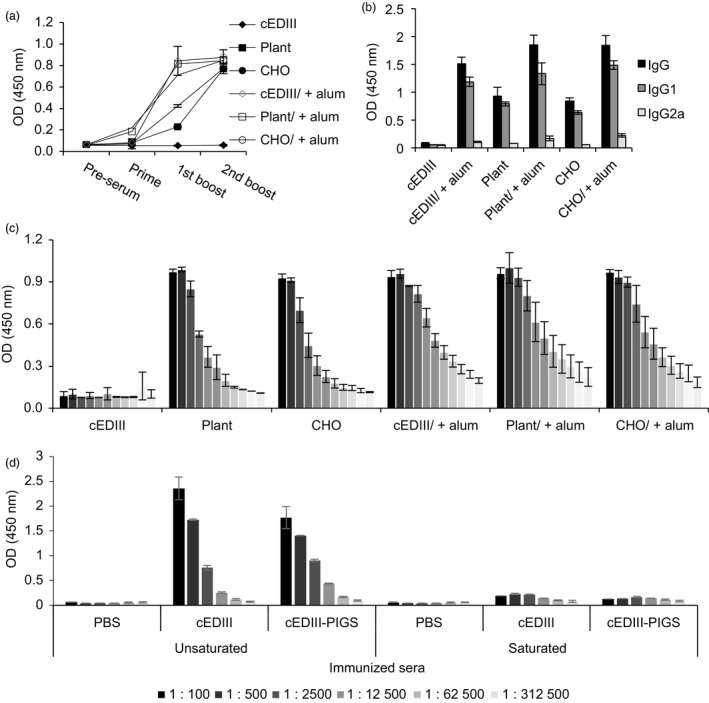
Serum IgG responses in mice immunized with cEDIII‐PIGS. (a) Kinetics of cEDIII‐specific IgG response in sera of mice during the immunisation protocol, detected by ELISA; shown are IgG responses from pooled sera of four mice (1:1000 dilution). (b) Analysis of IgG subtypes IgG1 and IgG2a in pooled sera from four immunized mice (1:1000 dilution). Results are shown as the means of triplicate measurements ± SD.(c) Relative IgG titres in fivefold serial dilutions (starting from 1:125) of sera from individual mice (*n* = 4), ± SD, at the end of the immunization protocol. (d) Specificity of the induced IgG response for dengue antigen; ELISA plates were coated with PBS, cEDIII or plant‐derived cEDIII‐PIGS and incubated with fivefold serial dilutions (starting from 1:100) of immune sera which were presaturated or not with recombinant cEDIII. Experiment performed twice with similar results.

Dengue‐specific IgG responses in mice were determined by ELISA after each immunization. cEDIII alone was not immunogenic, but with alum, a strong IgG response was observed. The cEDIII‐PIGS were immunogenic with and without alum, although peak responses were accelerated by the addition of alum (Figure 5a). The relative proportions of IgG1 and IgG2a were also determined (Figure 5b) and showing that IgG1 was produced at significantly higher levels than IgG2a. After third and final immunization, the end‐point titres for cEDIII‐PIGS were determined by serial dilutions. These were found to be 3.9 × 10^5^ (plant) and 7.8 × 10^4^ (CHO), respectively, while in the presence of alum, these were 1.5 × 10^6^ (for both plant and CHO‐derived cEDIII‐PIGS) (Figure 5c). Finally, we tested whether the IgG responses was directed exclusively to the cEDIII antigen or whether some reactivity may have been directed to the PIGS platform due to altered glycosylation in plants or the presence of neo‐epitopes. Immune sera detected both cEDIII and cEDIII‐PIGS in dilution‐dependent manner (Figure 5d). In contrast, when presaturated with recombinant cEDIII, the same sera could no longer detect either, thus confirming that all of the antibody reactivity was directed towards the cEDIII antigen alone.

### Dengue neutralization assay

**Figure 6:**
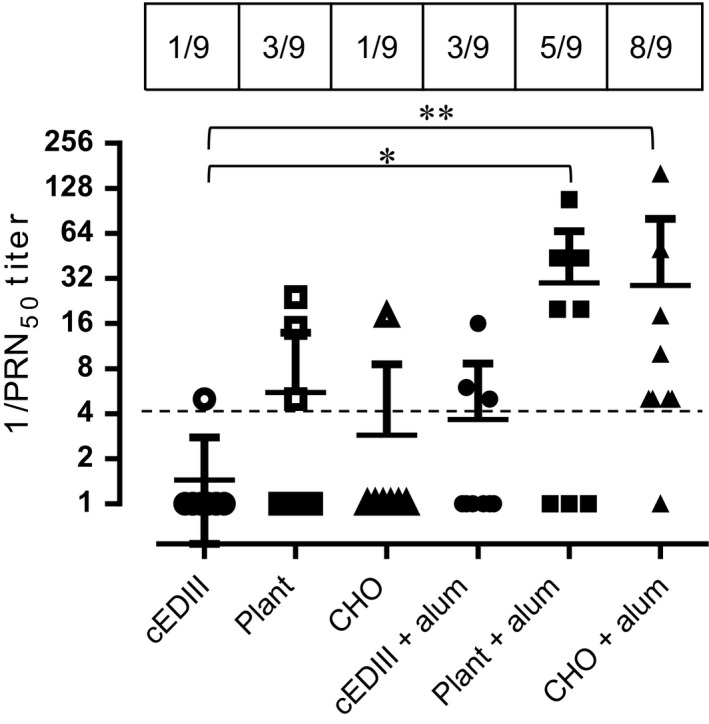
DENV 2 neutralization assay. Reciprocal values of mouse sera dilutions inducing 50% reduction in the viral plaques for dengue serotypes 2. Kruskal–Wallis nonparametric test was used to determine statistically significant differences between each of the test groups and the cEDIII group, with * and ** indicating *P* < 0.05 and 0.005, respectively. Perforated dashed line indicates the arbitrary cut‐off for neutralizing activity. Shown are combined results from two independent immunization experiments performed under similar conditions (*n* = 9).

To test the neutralizing potential of cEDIII‐PIGS‐induced antibodies, a neutralization assay using dengue virus serotype 2 was performed (Figure 6). While only one of the animals from the groups immunized with cEDIII alone or CHO‐derived cEDIII‐PIGS developed DENV2 neutralizing antibodies, neutralization was observed in 3/9 animals immunized with plant‐derived cEDIII‐PIGS. Inclusion of alum in the immunization regimen resulted in the sero‐neutralizing conversion of three animals from the cEDIII group but 5/8 and 8/9 animals from the plant‐ and CHO‐derived cEDIII‐PIGS groups, respectively.

### T‐cell proliferation and cytokine responses

**Figure 7:**
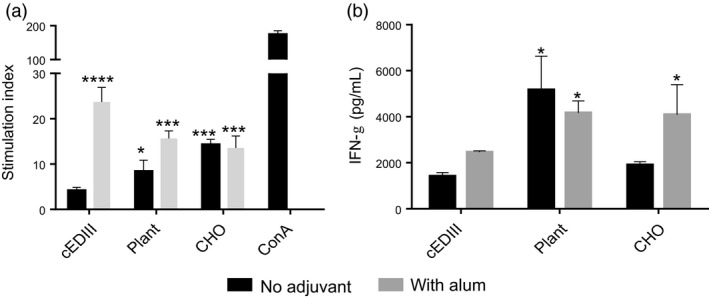
Analysis of cellular responses induced by cEDIII‐PIGS. (a) T‐cell proliferation in splenocyte cultures stimulated with *E.coli* expressed cEDIII. Shown are the stimulation indices obtained by dividing antigen‐induced with medium‐induced proliferation. (b) Detection of IFN‐γ in antigen‐stimulated splenocyte cultures; shown are the means of triplicate cultures of pooled splenocytes from four animals, + SD. Dark bars show IFN‐γ concentrations in cultures from animals immunized with cEDIII‐PIGS alone, whereas the grey bars represent animals immunized with cEDIII‐PIGS + alum. *, *P* < 0.05; **, *P* < 0.005; ***, *P* < 0.0005 ****, *P* < 0.0001 compared to cEDIII alone group.

T‐cell proliferation was determined in antigen‐stimulated splenocyte cultures by ^3^[H]‐thymidine incorporation. As can be seen in Figure 7a, there was no significant T‐cell proliferation in antigen‐alone immunized mice group, but the stimulation index was significantly higher for cEDIII‐PIGS immunized mice (eight for plant and 15 for CHO‐derived PIGS). In the presence of alum, all groups induced high level T‐cell proliferation, as indicated by indices above 10. Concanavalin A served as the positive control for cell proliferation. The analysis of IFN‐γ production in the supernatants from these splenocyte cultures indicated that cEDIII‐PIGS induced high levels of this Th1 cytokine with or without alum, although plant‐derived cEDIII‐PIGS were superior to CHO‐derived cEDIII‐PIGS (Figure 7b).

### cEDIII‐PIGS induce multifunctional T cells

**Figure 8:**
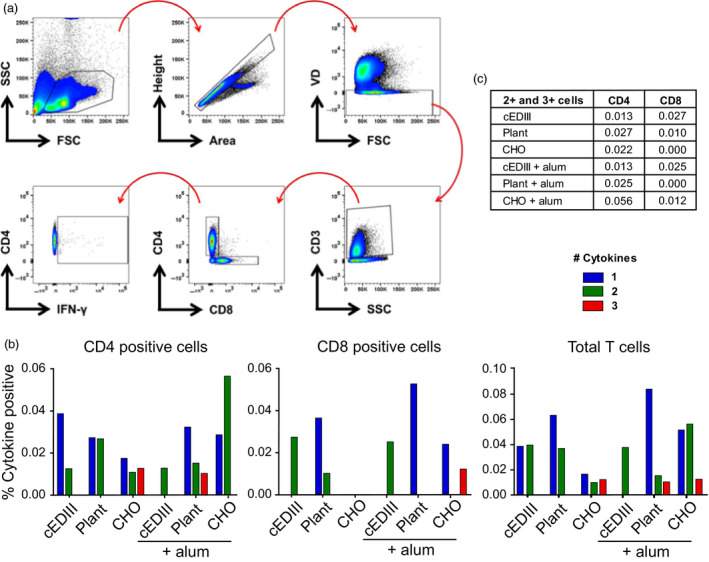
Analysis of polyfunctional T cells. (a) Gating strategy employed for analysis of CD4^+^ and CD8^+^ T‐cell subsets, based on cell size > single events > cell viability > T‐cell lineage > CD4 or CD8 positive > cytokine positive. A representative plot of IFN‐γ is shown; (b) the percentage of CD4^+^, CD8^+^ and total T‐cells positive for 1, 2 or 3 cytokines (of IFN‐ γ, IL‐2 and IL‐17). (c) sum total (%) of double‐ and triple‐positive CD4 and CD8 cells for each immunization group.

As cEDIII‐PIGS induced robust IFN‐γ responses in whole splenocytes, we next interrogated type 1 cytokine production more comprehensively at the cell‐specific level. Polyfunctional T cells are defined as cells capable of producing multiple effector cytokines and are a widely acknowledged biomarker of protection against several evolutionarily disparate pathogens (Seder *et al*., 2008). Hence, we assessed whether cEDIII‐PIGS could induce these cells after immunization. Splenocytes from each mouse immunization group were pulsed with cEDIII recall antigen, and flow cytometric analysis was used to measure intracellular cytokine responses (IFN‐ γ, IL‐2 and TNF‐α, i.e. cytokine ‘functions’) in CD4^+^ and CD8^+^ T cells, using the gating strategy as shown in (Figure 8a).

In CD4^+^ T‐helper cells, it was found that cEDIII immunization in the absence of adjuvant elicited a predominantly monofunctional response (0.038%) and a modest proportion of double‐positive cells (0.013%) (Figure 8c). Interestingly, plant‐derived cEDIII‐PIGS induced a lower percentage of monofunctional cells (0.027%) yet a higher percentage of double‐positive cells (0.027%) compared to native cEDIII. CHO‐derived cEDIII‐PIGS were found to induce a balanced profile of single‐ (0.017%), double‐ (0.010%) and triple‐positive (0.012%) cells. The addition of alum ablated the monofunctional cells in cEDIII‐immunized mice, yet produced the emergence of a triple‐positive population (0.010%) in the plant‐derived cEDIII‐PIGS group. In the CHO‐derived cEDIII‐PIGS group, the use of adjuvant caused a sharp increase in the percentage of double‐positive cells (0.056%).

In the CD8^+^ cytotoxic T‐cell compartment, responses were overall more muted compared to CD4^+^ T cells (Figure 8b). Immunization with cEDIII alone induced only double‐positive cells either with (0.027%) or without (0.025%) adjuvant, whereas the inclusion of adjuvant boosted monofunctional cytokine responses in the plant‐derived cEDIII‐PIGS group (0.036%–0.052%). For CHO‐derived cEDIII‐PIGS immunization, the adjuvant group had a high proportion of triple‐positive cells (0.012%) that was absent in other groups. Summation of total T‐cell polyfunctionality can be seen in (Figure 8b), showing that cEDIII‐PIGS immunization induced T cells with superior CD4+ T‐cell functionality (i.e. triple positive) compared to antigen alone.

## Discussion

Live, attenuated virus‐based dengue vaccines have been shown to be very immunogenic but their use in certain populations such as young children, the key group targeted for vaccination and immunocompromized individuals, remains uncertain. Coupled with suboptimal performance in recently completed human clinical trials, there is a need to develop alternative strategies, including potentially subunit vaccines. In the last year alone, several such vaccine candidates have been reported (Govindarajan *et al*., 2015; Manoff *et al*., 2015; Mareze *et al*., 2015; Poggianella *et al*., 2015; Suphatrakul *et al*., 2015) and a comprehensive recent review lists many others (Lam *et al*., 2015).

In this study, we evaluated the potential of a novel vaccination approach for dengue infection, based on self‐adjuvanting molecular forms designed to deliver multiple copies of the dengue glycoprotein E domain III to antigen presenting cells expressing immunoglobulin Fc receptors. Termed PIGS (Poly‐ImmunoGlobulin Scaffold), these molecules were modelled on the earlier work of Smith and Morrison (1994) but with the key modification that the antibody binding domains were replaced with antigen, resulting in polymeric antigen display. Indeed, this has been attempted before (Mekhaiel *et al*., 2011) with the incorporation of a *Plasmodium falciparum* antigen but without the desired outcome, as the polymers appeared less immunogenic than the monomers or dimers. In contrast, our study showed that cEDIII‐PIGS alone were highly immunogenic in mice, and even more so when alum was co‐administered. One reason could be that the functionality of these structures is dependent on the incorporated antigen but more likely, the difference could be ascribed to the modifications that were introduced, including the β‐strand of the C_H1_ domain and hinge region of IgG for flexibility and the substitution of proline 476 to threonine to make the last three or four residues of the murine IgG2a or human IgG1 C_H_3 domain identical to that of murine or human IgM C_H_4 domain.

Two versions of cEDIII‐PIGS molecules were generated using murine and human immunoglobulin sequences. As a key advantage of cEDIII‐PIGS used for antigen display is expected to be in relation to effector functions mediated through Ig heavy chain sequence, we demonstrated their binding to both the C1q component of complement and Fc receptor bearing cells. Binding to C1q is the first step in the antibody‐mediated activation of the complement, which leads to a cascade of reactions and formation of several complement components. While the C3b and C3d components opsonise pathogens and enhance their phagocytosis, the small components such as C3a, C4a and C5a act as inflammatory stimuli, contributing to the amplification of the immune response. The C1q binding ELISA demonstrated that cEDIII‐PIGS bound in a concentration‐dependent manner, unlike the IgG antibody alone, which only showed a moderate level of binding at the highest concentration tested. The ability of cEDIII‐PIGS to bind complement confirms the presence of polymeric structures and is also likely to contribute to immunogenicity *in vivo*. In another *in vitro* effector function assay, we tested whether cEDIII‐PIGS could bind to FcγRII in a Biacore assay or on macrophage cells by flow cytometry. As shown in Figure 4, the cEDIII‐PIGS polymers bound more effectively to immobilized FcγRII than monomers and also bound to J774 macrophage but not NIH‐3T3 cells. The macrophage cells express a range of Ig Fc receptors including FcγRI, FcγIIa and b, FcγRIIIa and b and binding by polymeric cEDIII‐PIGS points to increased uptake and antigen presentation. Taken together, these *in vitro* functional assays clearly demonstrate the potential of cEDIII‐PIGS to be delivered efficiently to the immune system in a self‐adjuvanting manner. This was confirmed by mouse immunization studies which revealed the mouse cEDIII‐PIGS to be highly immunogenic. They induced an antigen‐specific IgG end‐point antibody titre of 1:100 000 without adjuvant and 1:1 000 000 with alum, while immunization with the cEDIII antigen alone induced equivalent antibody titres only in the presence of alum. No significant differences were observed between the plants or CHO‐cell expressed cEDIII‐PIGS. The antibodies induced were then demonstrated to have dengue neutralizing activity. We tested neutralization of serotype 2 because it is the most difficult serotype to vaccinate against, as demonstrated by the suboptimal results for the Sanofi vaccine (Capeding *et al*., 2014; Villar *et al*., 2015), and also because it is the most predominant serotype in South‐East Asia. While sera from animals immunized with cEDIII antigen alone failed to neutralize, the sera from one‐third of mice immunized with plant‐derived cEDIII‐PIGS showed neutralizing activity. However, the majority of animals showed neutralizing activity following immunization with cEDIII‐PIGS in the presence of alum, suggesting that the neutralizing potential was dependent on higher antibody titres in those experimental groups. Even so, the observed DENV2 neutralizing activity was only moderate and we believe that this is due to the lack of neutralizing epitopes within cEDIII rather than the overall immunogenic potential of cEDIII‐PIGS. Further improvement could be achieved by modifying the antigen. To that end, we have already initiated studies in which the cEDIII had been extended to include the EDI/II hinge region from the envelope glycoprotein, which has previously been reported to contribute to its immunogenicity (Messer *et al*., 2014).

Interestingly, the analysis of the IgG subtypes suggested that cEDIII‐PIGS induced significantly more IgG1 (Th2) than IgG2a (Th1) (Figure 5b), but the analysis of splenocyte cultures indicated significant induction of the Th1 cytokine IFN‐γ (Figure 7b). IFN‐γ could play an important role in protection against dengue infection as suggested previously (Gil *et al*., 2012; Weiskopf *et al*., 2013). Indeed, the role of cellular immunity in protection against dengue infection has been recently re‐appraised (Rothman *et al*., 2015) as it seems increasingly plausible that more than just neutralizing antibodies are required to control viremia.

A growing body of evidence suggests that polyfunctional T cells play a pivotal role in protective immune responses to bacterial and viral pathogens (Seder *et al*., 2008). Whether these cells are ‘elite’ responders or simply a correlate of an advantageous immune state remains unclear. Regardless, it is apparent that the gross magnitude of an immune response does not always correlate with protection, but that the quality of the response may be a stronger indicator (Darrah *et al*., 2007). Crucially, it has been demonstrated that polyfunctional T cells exhibit direct protective qualities in the context of Dengue virus infection (Yauch *et al*., 2009). As we observed significant IFN‐γ production in splenocyte cultures from immunized mice, we investigated further whether this and other Th1 cytokines may be derived from polyfunctional T cells. Taken together, our data show that plant‐ and CHO‐derived cEDIII‐PIGS induced antigen‐specific cytokine responses from both CD4^+^ and CD8^+^ T cells, but with major qualitative differences compared to cEDIII alone. In CD4^+^ T cells, cEDIII immunization resulted in mostly monofunctional cells, but the mouse groups immunized with cEDIII‐PIGS displayed a high level of T‐cell polyfunctionality, containing populations of memory cells positive for three cytokines that were entirely absent from the former group. Moreover, in CD8^+^ T cells, cEDIII‐PIGS were able to induce triple‐positive cells. These data strongly suggest that cEDIII‐PIGS induce a superior immunological profile compared to native antigen, consisting of higher quality effector T cells.

Interestingly, the addition of alum to cEDIII‐PIGS appeared to decrease total cytokine responses for most groups. Further analysis of individual cytokines revealed that there was a consistent reduction in proportion of IFN‐γ^+^ cells in response to alum, particularly in the CD4^+^ compartment, which is consistent with its role as an inducer of Th2 polarisation (Brewer *et al*., 1999). Pertinently, this observation may also explain why alum augmented the cEDIII‐PIGS‐mediated induction of IgG1, as Th2 cytokines (i.e. IL‐4) are known to promote B‐cell IgG1 class switching and IFN‐γ antagonises this process.

Moving forwards, the results from murine studies justify progression to human IgG1‐based cEDIII‐PIGS and it is promising that these molecules have already been shown to assemble more efficiently than the murine version and that they are more stable. An approach that may enhance the Fc effector function of plant‐expressed human cEDIII‐PIGS is to engineer the glycans associated with the Ig heavy chain as was recently demonstrated that FcR binding of a plant antibody can be enhanced by removal of sugar residues that are commonly found in plant but not mammalian glycoproteins (Cox *et al*., 2006; Forthal *et al*., 2010).

We observed some differences in immunogenicity between the two forms of cEDIII‐PIGS from CHO cells and *N. benthamiana*, notably in splenocyte IFN‐γ production and T‐cell polyfunctionality profiles. These could have been due to distinct glycosylation profiles in plants and animal cells. However, we believe that the plant expression system offers considerable potential for producing vaccines on a large scale at a lower cost. This would be particularly important for developing a dengue vaccine for which the primary market is the developing world. This proof‐of‐principle study provides a clear indication of the potential of the ‘PIGS’ technology as a generic vaccine delivery platform against dengue and possibly other infectious diseases.

## Methods

### Gene construction for cEDIII‐PIGS

Two construct sets were made, one using murine immunoglobulin sequence—for preliminary evaluation and the other using human immunoglobulin sequence—for subsequent product development. Genes encoding the mouse IgG2a‐Fc (Fragment crystallizable) and human IgG1‐Fc scaffolds were codon‐optimized to *Nicotiana tabacum* and *H. sapiens* and synthesized by Life Technologies, UK. Provided in the pMA‐T vector, the sequences include *att*B1 and *att*B2 sites for recombinational cloning into Gateway vectors. A Kozak consensus sequence (ACCATGG) allows efficient translation in both plant and mammalian expression systems. The [*ATGC*]*BsaI‐BsaI*[*GCTT*] cassette permits cloning of genes of interest between the leader sequence and poly‐immunoglobulin G Fc scaffold (PIGS) sequences. This results in the fusion of the gene product of interest to the N terminus of the Fc polypeptide. To confer additional flexibility, the scaffold sequence includes the last 9‐10 amino acid residues (corresponding to the final β‐strand) of the CH_1_ domain and hinge region of IgG. Addition of either murine or human mu‐tail piece (μtp) sequence at the 3′ terminus results in polymers with enhanced effector functions (Smith *et al*., 1995). A Pro^476^ → Thr substitution (Kabat numbering) was included at the end of the murine IgG2a and human IgG1 C_H_3 domain. The rationale for this substitution was to make the last three or four residues of the murine IgG2a or human IgG1 C_H_3 domain identical to the last three or four residues of mouse or human IgM C_H_4 domains. The human sequence includes a Cys^230^ → Ser substitution in the hinge region of IgG1 to remove the Cys residue that would naturally make a disulfide bond with the terminal Cys residue of the light chain. In the absence of a light chain, this substitution is intended to avoid unpaired Cys residues in PIGS fusion proteins.

DNA encoding the 103 amino acid long consensus dengue envelope domain III, cEDIII, was amplified using Phusion^®^ High‐Fidelity DNA polymerase (NEB) with forward, BsmBI‐EDIII‐F (5′‐ ttttCGTCTC *A*ATGCAAGGGCATGTCCTACGCTATG‐3′) and reverse primers, BsmBI‐EDIII‐R (5′‐ ttttCGTCTC *A*AAGCTGAAGAACCCTTCTTGAACCAGTT‐3′), and the plasmid DNA, pMYV657 (Kim *et al*., 2013) as the template. The cEDIII PCR fragment‐bearing *BsmBI* site which is compatible with *Bsa*I sites for cloning was then cloned into pMA‐T plasmid containing either tobacco or human codon‐optimized murine and human version of PIGS molecules using *BsaI* restriction enzyme site. For expression in plants, the construct was then digested by *NcoI* and *XbaI* restriction enzymes and subcloned into the pTRAk.2 vector which is derivative of pTRAk (Sack *et al*., 2007).

### Expression of cEDIII‐PIGS in tobacco cells

For expression of cEDIII‐PIGS constructs in plants, both wild type and a ΔXF variant of *N. benthamiana* were used in this study. The ΔXF variant produces glycoproteins that resemble mammalian glycoproteins more closely than the wild‐type plant (Strasser *et al*., 2008) and for that reason data for functional analysis of cEDIII‐PIGS presented in this study are shown for proteins derived from ΔXF plants. The agro‐infiltration of plant leaves was performed with tobacco codon optimized cEDIII‐PIGS constructs alone or in combination with the J chain construct (to generate hexameric or pentameric molecules, respectively). A 10‐mL starting culture of the transformed agrobacteria was grown for 2 days and used to inoculate a 200‐mL culture in YENB medium. After 1 day, the culture was centrifuged at 4500 g for 15 min and agrobacteria were re‐suspended in the infiltration buffer (10 mM MES, 10 mM MgCl_2_) to achieve an OD_600_ of 0.2. The agroinfiltration was performed as described previously (Kim *et al*., 2015).

### Expression of cEDIII‐PIGS in mammalian cells

One day before transfection, Chinese Hamster Ovary (CHO) K1 cells (ATCC) were seeded in 24‐well tissue culture plates in culture medium [Dulbecco's modified Eagle's medium supplemented with 4 mm_L_‐glutamine, 100 U/mL penicillin and 0.1 mg/mL streptomycin (Sigma), and 10% FBS]. For the generation of codon‐optimized cEDIII‐PIGS producing cell lines, the cells were transfected with the expression vectors, encoding both murine and human versions of cEDIII‐PIGS/pEF‐DEST51 using the FuGENE^®^6 transfection protocol (Promega). Three days post‐transfection, adherent cells were harvested and plated in selective medium (containing 7.5 μg/mL of blasticidin) for 1 week, until all non‐transfected cells were eliminated. The remaining cells were plated in 96‐well plates in serial dilutions to allow for selection of single clones and maintained in medium with 2.5 μg/mL of blasticidin. Supernatants from these clonal cultures were screened for expression of recombinant protein by sandwich enzyme‐linked immunosorbent assay and by immunoblotting.

### Immunization of mice

For immunization with cEDIII‐PIGS, 12‐ to 16‐week‐old inbred male and female Balb/c mice were used. The mice were kept under defined environmental conditions (12:12‐h light:dark cycle, 19–21 °C, 55% relative humidity, pathogen‐free). Five mice per group were immunized subcutaneously with 15 μg of plant or mammalian‐derived cEDIII‐PIGS (equivalent to 4.5 μg of cEDIII) in 100 μL, with or without aluminium hydroxide gel (Sigma). Two or three (in two independent experiments) additional boost immunisations were performed at 2‐week intervals. Mice were bled after each immunization step by tail vain, to monitor the antibody titres. Two weeks after the final immunization, the mice were sacrificed and bled by cardiac puncture and the spleens were collected for analysis of cellular immune responses.

### Humoral response

Specific humoral responses in sera of mice immunized with bacterially expressed cEDIII alone or plant and CHO‐derived cEDIII‐PIGS were tested by ELISA. 10 μg/mL of recombinant cEDIII protein was coated on Nunc Maxisorp 96‐well ELISA plates and incubated overnight at 4 °C. After blocking, immunized sera were added in fivefold serial dilutions and incubated for 2 h at 37 °C. The wells were washed with PBS‐T and peroxidase‐conjugated detection antisera [anti‐mouse IgG, anti‐mouse IgG1 (The Binding Site), or anti‐mouse IgG2a‐peroxidase (Bio‐Rad)] were added at 1:1000, and incubated as before. Finally, the ELISA plates were developed by addition of the OPD peroxidase substrate (Sigma). In a further modification of the ELISA protocol, immune sera were preincubated with bacterially expressed cEDIII (10 μg/mL) and were then used to test reactivity in cEDIII‐ or cEDIII‐coated plates.

### Dengue virus neutralization assay

Neutralizing titres of the immune sera were determined as 50% plaque reduction of viral infectivity against Dengue 2 (New Guinea C strain, VR‐1584 ATCC). Twofold serum dilutions in Hanks balanced salt solution were prepared and incubated with 50 PFU of the virus for 1 h at 37 °C. The remaining infectivity was determined by inoculation of BHK‐21 cells in the presence of overlay medium (MEM, 3% carboxymethylcellulose). Plaques were developed at 5 or 7 days post‐infection with a solution of naphthol blue‐black after cell fixation with 10% formaldehyde. Neutralizing titres were estimated by comparison with irrelevant sera, as the highest dilution which causes 50% reduction of plaques.

### Intracellular cytokine staining and flow cytometry

To measure T‐cell cytokine production and polyfunctionality, 1.5 million splenocytes were pulsed with 5 μg/mL cEDIII in duplicates in the presence of brefeldin A (5 μg/mL; Sigma‐Aldrich, UK) for 4 h. Cells were then incubated with a viability dye (VD) (eFluor^®^ 780; 1:1000 dilution, eBioscience, UK) for 30 min at 4 °C, washed and fixed in 100 μL BD Cytofix (Becton Dickinson, UK) for 30 min at 4 °C and then washed and permeabilized with flow cytometry buffer (PBS, 0.5% BSA, 0.1% sodium azide; all from Sigma‐Aldrich) containing 0.5% saponin (Sigma‐Aldrich) for intracellular cytokine staining (ICS) using eight‐colour polychromatic flow cytometry. As negative and positive controls, brefeldin A‐treated cells from each group of mice were either left unstimulated or treated with an activation cocktail of phorbol myristate acetate (PMA)/ionomycin (200 ng/mL and 1 μg/mL; Sigma‐Aldrich, UK), respectively.

For ICS, cells were stained for 45 min at 4 °C in flow cytometry buffer (+0.5% saponin) containing pretitrated antibodies (Biolegend, UK) specific for CD3ε (FITC, 145‐2C11), CD4 (PerCP‐Cy5.5, GK1.5), CD8α (Alexa Flour^®^ 700, 53‐6.7), IFN‐γ (PE‐Dazzle**™** 594, XMG1.2), IL‐2 (PE, JES6‐5H4), IL‐17A (PE‐Cy7, TC11‐18H10.1) and TNF‐α (APC, MP6‐XT22). Cells were then washed extensively and acquired on a BD LSR II instrument within 6 h of staining. For panel compensation, UltraComp eBeads^®^ (eBioscience, UK) were used according to the manufacturer's instructions.

### Polyfunctional T‐cell analysis

All ICS data analysis was performed using FlowJo v.10, MS Excel 2010 and GraphPad Prism v.6 software, using a previously described protocol (Roederer *et al*., 2011). Cells were assessed using the following gating strategy: lymphocyte gate (FSC/SSC) > singlets (area/height) > viable cells (VD^neg^) > TCR^pos^ (CD3^+^) > T helper (CD4^+^) or cytotoxic T cells (CD8^+^). Gating boundaries were determined by FMOs and biological controls. A Boolean gating platform was used to assess T‐cell polyfunctionality. For all analyses, background cytokine production in the negative control samples was subtracted from the cEDIII‐pulsed cells for each cytokine combination. A cytokine signal threshold of 0.01% was applied to background‐subtracted data and all subthreshold values were set to zero.

### Statistical analysis

The cell culture‐based assays were performed in triplicates, and the values (from a representative experiment of typically three performed, with the exception of ICS experiment, which was performed only once) are shown as the mean ± standard deviation. For immunisation experiments, four or five animals were used per group (in two independent experiments). For all assays which had more than two experimental variables, Dunnett's multiple comparison test was used, with the exception of DENV 2 neutralization assay, for which Kruskal–Wallis nonparametric test was used. GraphPad Prism v.6 software was used for statistical analysis, and the differences were significant when the *P* value was 0.05 or less.

## Supporting information


**Table S1** Contains amino acid sequences of cEDIII and PIGS (polymeric IgG scaffold) molecules.
**Data S1** Additional methodology description, including: expression vector for *Nicotiana benthamiana*, expression vector for mammalian cells, recombinant cEDIII expression, detection of cEDIII‐PIGS by electrophoresis and Western blotting, extraction and purification of cEDIII‐PIGS, binding of cEDIII‐PIGS to complement C1q, binding of cEDIII‐PIGS to antigen‐presenting cells, Biacore measurements, IFN‐γ ELISA, cellular responses and HPLC fractionation of cEDIII‐PIGS.
