# Supplementary material for: Molecular engineering and plant expression of an immunoglobulin heavy chain scaffold for delivery of a dengue vaccine candidate
Source: Plant Biotechnol J. 2017 Jul 15;15(12):1590–601. doi: 10.1111/pbi.12741 (PMC5698049; doi:10.1111/pbi.12741)
Supplement: Supplementary file 1 — Table S1 Contains amino acid sequences of cEDIII and PIGS (polymeric IgG scaffold) molecules. Data S1 Additional methodology description, including: expression vector for Nicotiana benthamiana, expression vector for mammalian cells, recombinant cEDIII expression, detection of cEDIII‐PIGS by electrophoresis and Western blotting, extraction and purification of cEDIII‐PIGS, binding of cEDIII‐PIGS to complement C1q, binding of cEDIII‐PIGS to antigen‐presenting cells, Biacore measurements, IFN‐γ ELISA, cellular responses and HPLC fractionation of cEDIII‐PIGS. [file PBI-15-1590-s001.docx]

**Molecular engineering and plant expression of an immunoglobulin heavy chain scaffold for delivery of a dengue vaccine candidate**

**Mi-Young Kim^1,2^, Craig Van Dolleweerd^1^, Alastair Copland^1^, Matthew John Paul^1^, Sven Hofmann^1^, Gina R. Webster^1^, Emily Julik^3^, Ivonne Ceballos-Olvera^3^, Jorge Reyes-del Valle^3^, Moon-Sik Yang^2^, Yong-Suk Jang^2^, Rajko Reljic^1^* and Julian K. Ma^1^***

1. Institute for Infection and Immunity, St George’s University of London, UK
2. Department of Molecular Biology and The Institute for Molecular Biology and Genetics, Chonbuk National University, Jeonju 54896, Korea
3. School of Life Sciences, Arizona State University, Tempe, AZ, USA

**Data S1**

**Expression vector for *Nicotiana benthamiana***

For expression in tobacco, the pTRAk.2 construct was transformed into agrobacterium strain GV3101 containing pMP90RK helper plasmid, by electroporation and selected on YENB medium (7.5g of Bacto-yeast Extract, 8g of Nutrient broth, pH 7.5), containing 50 μg/mL each of carbenicillin and rifampicin. The plasmid containing human J chain for co-expression with cEDIII-PIGS to facilitate the assembly of PIGS into the polymer in plants was described previously ^[46](#_ENREF_46)^.

**Expression vector for mammalian cells**

For expression in mammalian cells, the attB1-leader sequence-cEDIII-PIGS-attB2 cassette of mammalian expression optimized cEDIII-PIGS was first recombined with the donor vector (pDONR/Zeo, Life technologies) and then with the destination vector (pEF-DEST51, Life Technologies), using the Gateway Technology protocol (Life Technologies).

**Recombinant cEDIII expression**

Recombinant cEDIII antigen alone was used as an immunisation control in this study. The cEDIII gene obtained by BamHI-KpnI digestion of pMYV657 [^44^](#_ENREF_44) was cloned into the same sites of pQE30 (QIAGEN, Hilden, Germany) carrying the gene for an N-terminal His-tagged protein, to yield cEDIII/pQE30. The recombinant plasmid was transformed into *E. coli* M13 competent cells (QIAGEN) and expression was induced by addition of 1 mM isopropyl β-D-1-thiogalactopyranoside (IPTG) for 5 h at 37˚C. The bacteria were harvested by centrifugation at 5000*g* for 15 min and the pellet was re-suspended in buffer (8 M urea, 100 mM NaCl, 20 mM HEPES, pH8.0) and sonicated. After centrifugation the recombinant protein was purified using Ni-NTA chromatography (QIAGEN), according to manufacturer’s directions. LPS was minimised using polymyxin B-agarose chromatography (Sigma, USA).

**Detection of cEDIII-PIGS by electrophoresis and Western blotting**

To confirm the expression of cEDIII-PIGS in plant extracts or supernatant of CHO cell culture, samples were run on 4-12 % or 12 % Bis-Tris gels (Life Technologies) using NuPAGE® MOPS SDS Running Buffers (Life Technologies). Following electrophoresis, gels were stained with InstantBlue (Expedeon) or subjected to Western blot analysis. The blotted membrane was blocked for 30 min with 5 % (w/v) non-fat dried milk in PBS and incubated overnight with peroxidase-conjugated anti-mouse IgG or anti-human IgG antiserum (1:1000 dilution; The Binding Site) for detection of the IgG Fc portion, or with mouse anti-dengue virus monoclonal antibody (1:2500 dilution; Bio-Rad AbD Serotec) followed by anti-mouse IgG (light chain specific) peroxidase-conjugated antiserum (1:1000, Jackson ImmunoResearch). The blots were washed with PBS/0.01 % Tween-20 (PBST) and developed using the ECL Plus Western blotting detection system (GE Healthcare).

To visualise high molecular weight structures, the purified recombinant proteins were separated on 3-8 % Tris-Acetate gels using NuPAGE® Tris-Acetate SDS Running Buffer or 3-8 % NativePAGE™ Bis-Tris gels (Life Technologies), followed by fixation (H_2_O, methanol, and acetic acid in a ratio of 50/40/10) and Coomassie Blue R250 staining.

**Extraction and purification of cEDIII-PIGS**

To extract plant derived cEDIII-PIGS, infiltrated tobacco leaves were homogenized by a blender with 2 volumes PBS buffer and the crude extracts filtered through Miracloth (Calbiochem) and centrifuged at 18000 rpm for 30 min in a ROTINA 48R centrifuge (Hettich Zentrifugen). The supernatant was sterilized through a 0.22 μm filter before applying to a protein A agarose affinity column (Sigma). After extensive washing with PBS, the bound protein was eluted in 0.1 M glycine-HCl, pH 2.7, and the fractions neutralized by addition of 1 M Tris base (pH unadjusted).

To purify CHO derived cEDIII-PIGS, the culture supernatant was filtered through a 0.22 μm filter and applied onto an anti-mouse or human IgG Agarose affinity column (Sigma, A6531 or A3316) and eluted as described above.

The protein content in fractions was determined by measuring optical density at 280 nm. The pooled fractions were concentrated by ultrafiltration using an Amicon ultra-4 Centrifugal Filter Unit (Millipore, Billerica, MA) and dialyzed against PBS.

**Binding of cEDIII-PIGS to complement C1q**

To confirm the binding affinity of cEDIII-PIGS to complement C1q protein, 10 μg/mL of human C1q (Calbiochem) was coated onto ELISA plates and incubated overnight at 4^o^C. After blocking in 5% non-fat dry milk protein solution in PBS, 2-fold serial dilutions of samples were added and incubated at 37^o^C for 2 h. The previously described recombinant immune complex mimics (ICM) [^24^](#_ENREF_24) and a mouse IgG2a antibody (Sigma) were used as the positive and negative control. Peroxidase-conjugated anti-mouse IgG antiserum was used as the detection antibody. The peroxidase reaction was developed by adding 50 μL of OPD peroxidase substrate solution (Sigma) to each well. The colour reaction was stopped by addition of 25 μL/well of 2 M H_2_SO_4_ and the absorbance was determined at 450 nm using a Sunrise plate reader (Tecan, UK).

**Binding of cEDIII-PIGS to antigen-presenting cells**

To test the capacity of cEDIII-PIGS to bind to antigen-presenting cells, J774 macrophage cells (ATCC) were maintained in complete RPMI medium [RPMI 1640 + 2mM L-glutamine + 10% Foetal Bovine Serum (FBS)] in 5 % CO_2_ at 37 °C. NIH-3T3 mouse fibroblast cells (ATTC) grown under similar conditions were used as a negative control. The cells growing at 80 % confluence were detached using dissociation buffer (Invitrogen), pelleted by centrifugation at 1,000 rpm for 5 min and resuspended in binding buffer (3 % BSA in PBS and 0.05 % sodium azide). 1 million cells in 100 μL buffer were presaturated or not with 2 μg/ml of Fc block antibody (anti-CD16/32, Biolegend) and were incubated on ice for 2 h with 100 μg/mL of cEDIII-PIGS. Unbound protein was removed by washing 3 times with 3 mL of binding buffer. 7.5 μL of secondary antibody [anti-mouse IgG-FITC antiserum (The Binding Site)] was added and the cells incubated for a further 1 h on ice. After washing as before, cells were resuspended in 500 μL of binding buffer and analysed for green fluorescence in a Becton-Dickinson flow cytometer. Secondary antibody alone was used for background staining.

**Biacore measurements**

The avidity of the cEDIII-PIGS complexes in binding to murine CD16 was measured using a Biacore X100 instrument (GE Life Sciences, Little Chalfont, UK). Briefly, 12000 RU of an anti-his antibody was immobilized on both flow channels of a CM5 sensor chip using amine coupling chemistry (His capture kit, GE Life Sciences). For each sample, recombinant murine CD16 containing a His tag (1960-FC, R&D systems) diluted to 2 μg/ml in HBS-EP+ running buffer was captured on Fc2 to a level of 750 RU. Samples (10 μg/ml) were injected over both flow channels with a contact time of 80s and a flow rate of 30ul/min, and dissociation monitored for 300s. Regeneration of the surface was achieved by a 30 s pulse of 10mM glycine pH 1.5.

**IFN-γ ELISA**

For IFN-γ ELISA, 50 μL/well of the capture antibody in 1x Coating buffer was coated in ELISA plates. The experimental procedure described by the manufacturer (Mouse Th1/The ELISA Ready-SET-Go kit; affymetrix eBioscience, USA) was then followed. The concentration of secreted IFN-γ in splenocyte cultures was calculated from the standard curve performed within the assay.

**Cellular responses**

To obtain splenocytes, spleens were extracted aseptically from immunized mice, pooled and homogenised in 10 mL of complete RPMI medium (Sigma) using a 5-ml syringe plunger. The tissue was squeezed through a 70 μm cell strainer (BD FalconTM). The released cells were spun and the pellet resuspended in RPMI medium. To eliminate red blood cells, the pelleted cells were incubated with ACK lysing buffer (Gibco) for 3 min at 37^o^C and washed two times with 25 ml complete medium. Triplicate cultures were seeded into 96 well U-bottom plates at a density of 3x10^5^ cells/well, in 200 μL medium and stimulated with 10 μg/mL of cEDIII antigen. The medium alone and Concanavalin A (ConA) at 5 μg/mL were used as the negative and positive control for splenocyte proliferation, respectively. After incubation of cells for 48 h at 37 ^o^C, a 100 μL of supernatant was removed for Th1/Th2 cytokine ELISA assay. For T-cell proliferation assay, the cultures were pulsed with 1 mCi/well of ^3^[H]-thymidine (GE Healthcare) and incubated for a further 24 h before harvest. At harvest, cells were transferred onto a glass fiber filter (Wallac) using a TOMTEC harvester and fixed onto a scintillator sheet (MeltiLexTM A, Perkin Elmer), prior to determining counts per minute (CPM) in the scintillator counter (Wallac).The stimulation indices were calculated as the ratios between specific and medium induced proliferation for each immunisation group.

**HPLC fractionation of cEDIII-PIGS**

Native size-exclusion chromatography (SEC) was carried out on a Shimadzu LC2010AHT system (Milton Keynes, UK) equipped with a UV-detector using a Yarra 3u SEC-4000 column (7.8 x 300 mm, 3 µm particle size; Phenomenex, Cheshire, UK). Chromatographic separation was achieved by isocratic elution using standard phosphate buffered saline (PBS) at a flow rate of 1 ml/min. Samples were prepared in PBS at a concentrations of 1 mg/ml. For analytical SEC 5-40 µg protein in 20 µl injection volume were loaded, whereas for preparative fractionation 100-200µg protein in 100 µl injection volume were injected in repetitive cycles. Fraction collection was performed manually. Proteins were detected at 280 nm. Data acquisition and post-run analysis were controlled by LabSolutions (Version 5.75 SP2). As reference, the BioRad GFC Standards were used and molecular weights of different cEDIII-PIGS fractions obtained by comparison of the respective elution times.

**Table S1. Amino acid sequences of cEDIII and PIGS (polymeric IgG scaffold)**

|  | Mouse IgG2a | Human IgG1 | Note |
| --- | --- | --- | --- |
| Human Leader | MELGLSWIFLLAILKGVQC | |  |
| cEDIII | KGMSYAMCTGKFKLEKEVAETQHGTILIKVKYEGDGAPCKIPFEIQDVEKKHVNGRLITANPIVTDKESPVNIEAEPPFGDSYIVIGVGDKALKLNWFKKGSS | |  |
| C_H_1 domain | ASSTKVDKKI | ASNTKVDKKV |  |
| Hinge | EPRGPTIKPCPPCKCP | EPKSSDKTHTCPPCP | S: Cys^230^ 🡺 Ser |
| C_H_2 domain | APNLLGGPSVFIFPPKIKDVLMISLSPIVTCVVVDVSEDDPDVQISWFVNNVEVHTAQTQTHREDYNSTLRVVSALPIQHQDWMSGKEFKCKVNNKDLPAPIERTISKPK | APELLGGPSVFLFPPKPKDTLMISRTPEVTCVVVDVSHEDPEVKFNWYVDGVEVHNAKTKPREEQYNSTYRVVSVLTVLHQDWLNGKEYKCKVSNKALPAPIEKTISKAK |  |
| C_H_3 domain | GSVRAPQVYVLPPPEEEMTKKQVTLTCMVTDFMPEDIYVEWTNNGKTELNYKNTEPVLDSDGSYFMYSKLRVEKKNWVERNSYSCSVVHEGLHNHHTTKSFSRTTGK | GQPREPQVYTLPPSRDELTKNQVSLTCLVKGFYPSDIAVEWESNGQPENNYKTTPPVLDSDGSFFLYSKLTVDKSRWQQGNVFSCSVMHEALHNHYTQKSLSLSTGK | T: Pro^476^ 🡺 Thr |
| µtp | PTLYNVSLIMSDTGGTCY | PTLYNVSLVMSDTAGTCY |  |
